# Post-traumatic Total Hip Arthroplasty After Acetabular Fractures: Benefits of the Hardinge Approach

**DOI:** 10.7759/cureus.63537

**Published:** 2024-06-30

**Authors:** Humza S Shaikh, Saad Mohammad, Tyler D Petersen, Steven Cotman, Peter A Siska

**Affiliations:** 1 Department of Orthopaedic Surgery, University of Pittsburgh Medical Center, Pittsburgh, USA; 2 Department of Orthopaedic Surgery, The Ohio State University Wexner Medical Center, Columbus, USA

**Keywords:** reconstruction hip and knee surgery, acetabulum fracture fixation, hardinge approach, hip total arthroplasty, post-traumatic hip arthritis, fracture of acetabulum

## Abstract

Post-traumatic arthritis is a common sequelae after undergoing open reduction and internal fixation (ORIF) of acetabular fractures. This often necessitates conversion to total hip arthroplasty (THA) to help alleviate pain and improve function for these patients. Unfortunately, dislocation rates for post-traumatic THA have been alarmingly high especially when the posterior approach has been used. In the setting of prior soft tissue disruption, the theoretical risk of dislocation is even greater. Conversely, the lateral or the abductor-split approach (Hardinge) is associated with decreased dislocation rates. In this retrospective case series, we evaluated the dislocation rate of the Hardinge approach on patients who underwent THA after developing post-traumatic arthritis after acetabulum ORIF. All patients who matched CPT code 27132 (Repair, Revision, and/or Reconstruction Procedures on the Pelvis and Hip Joint), from January 2009 to December 2019, and treated by the senior author, were pulled from the electronic medical record at the University of Pittsburgh Medical Center. Thirty-one of the resultant 110 were treated with THA for post-traumatic arthrosis through a lateral, abductor-splitting Hardinge approach and met the inclusion criteria for further study. Our case series involves 31 patients who underwent post-traumatic THA through a Hardinge approach: the mean age at the time of index acetabular ORIF is 48.5 years, the mean age at the time of THA is 53.5 years, and the mean interval between ORIF and ultimate THA was five years. The mean length of follow-up after THA was 22.4 months. Overall, patients did well with an all-cause revision rate of 9.7%, with no revision performed for loosening of either the acetabular or femoral component. One patient developed an infection. No patient in our group sustained a dislocation, and all implants were stable without evidence of radiographic loosening at the final follow-up. This study found satisfactory results with patients undergoing THA via lateral or abductor split approach (Hardinge) for post-traumatic arthritis after acetabular ORIF. The use of a Hardinge approach for post-traumatic reconstruction of the hip may be protective against dislocation without increasing baseline risks in this difficult patient population.

## Introduction

Despite modern fracture management techniques that yield near-anatomic reduction of complex acetabular fracture, post-traumatic arthrosis continues to pose a major challenge [1]. Greater than 20% of patients with acetabular fractures treated with open reduction and internal fixation (ORIF) go on to develop significant post-traumatic arthrosis necessitating conversion to total hip arthroplasty (THA) [2-4]. Unfortunately, the risk of complication after THA for post-traumatic arthrosis is significantly higher when compared to primary THA [5,6]. Common complications include infection, loosening, heterotopic ossification, periprosthetic fracture, and dislocation. In fact, dislocation rates for post-traumatic THA have been reported upwards of 11% [7].

As in the case of primary total hip arthroplasties, the most common approach for post-traumatic reconstruction remains a posterior exposure [8,9]. In the setting of prior posterior column injuries, surgeons are often able to utilize the same exposure as the index ORIF. Unfortunately, even in the absence of prior trauma, dislocation rates associated with a posterior approach to the hip have been reported at around 1% with capsular repair, but with over an eightfold greater relative risk of dislocation in the absence of posterior soft tissue repair [10]. In the setting of revision posterior exposure, prior scarring may impede posterior soft tissue reconstruction, placing post-traumatic THAs at a much greater theoretical risk of dislocation.

Conversely, the lateral, abductor-split approach (Hardinge) has reported dislocation rates of less than 1% [10]. The purpose of this paper is to determine whether the use of the Hardinge approach would reduce rates of dislocation after post-traumatic THA in patients who have undergone a previous Kocher approach for ORIF of an acetabulum fracture.

## Materials and methods

This is a retrospective case series approved by the institutional review board (IRB 19110171) of the University of Pittsburgh. We accessed the electronic medical records of patients at the University of Pittsburgh Medical Center using the CPT code 27132 (Repair, Revision, and/or Reconstruction Procedures on the Pelvis and Hip Joint), who underwent the procedure from January 2009 to December 2019. All patients were treated by the senior author, a fellowship-trained orthopedic trauma surgeon. Inclusion criteria consisted of patients undergoing revision THA using the Hardinge approach for post-traumatic arthritis after acetabular ORIF. All other patients undergoing THA for any other reason or a different approach were excluded from the study.

**Figure 1:**
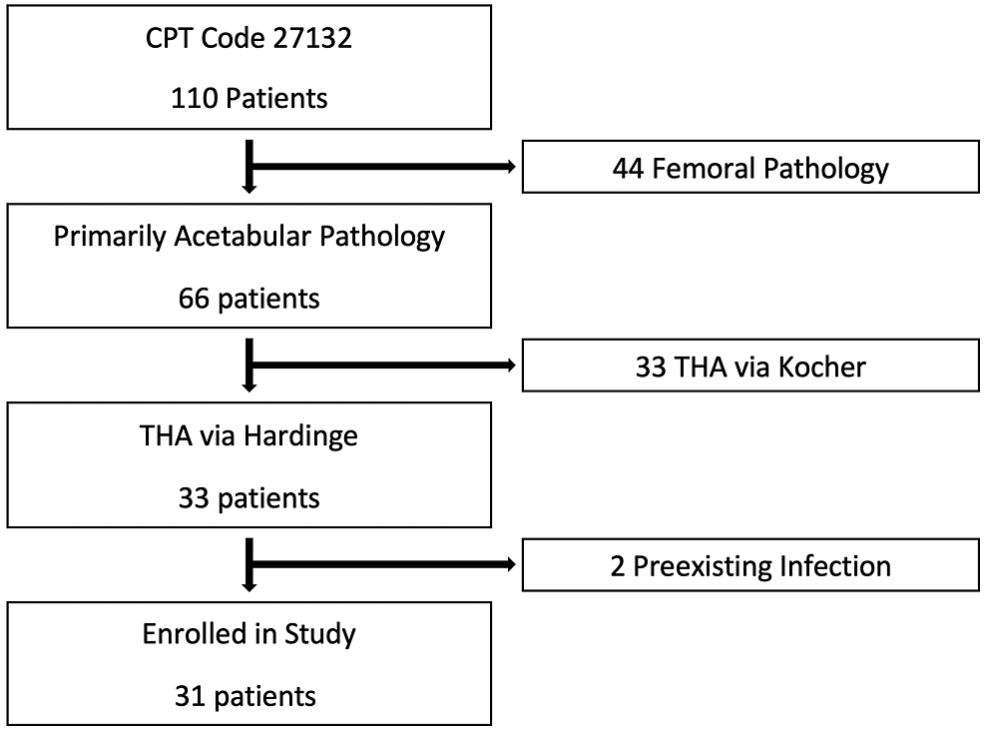
Flow chart of the patient selection Current Procedural Terminology (CPT), Total Hip Arthroplasty (THA)

The resultant 110 patient records were reviewed, including patient notes, intra-operative records, and all pre- and post-operative imaging. Of these, a subset of 31 who were treated with THA for post-traumatic arthrosis through a lateral, abductor-splitting Hardinge approach, met inclusion criteria for further study. Our data collection consisted of extensive chart review, which included patient age, date of index acetabular reconstructive surgery, date of total hip arthroplasty, date of final follow-up, and peri-operative/post-operative complications, including outcomes following revision surgery (Figure 1).

## Results

In total, 31 patients were included in this case series, 22 men and nine women. There were no dislocations, no post-operative neurologic complications, and no evidence of loosening in this cohort at any time point during the follow-up period.

The mean age at the time of index acetabular ORIF was 48.5 years, and the mean age at the time of THA was 53.5 years. The mean interval between ORIF and ultimate THA was 5.0 years. The mean length of follow-up after THA was 22.4 months (Table 1).

**Table 1:** Follow-up and complications after post-traumatic total hip arthroplasty Open Reduction Internal Fixation (ORIF), Total Hip Arthroplasty (THA) * Age at the original acetabular ORIF, ** Metal on the metal THA with elevated ion levels, *** Intra-operative greater trochanteric fracture

	Age at Surgery* (years)	Femur/Acetabulum	Time from ORIF to THA (months)	Length of follow-up (months)	Revised (Y/N)	Complications
	20.4	F/A	202.5	115.2	Y	MoM reaction**
	23.9	A	87.9	12		
	25.7	A	151.3	44.4	Y	Component malpositoin
	26	A	17.2	78	Y	Infection
	32.9	A	159.3	3.4		
	34.6	A	13.2	24.8		
	35	A	108.5	1.1		
	39.3	A	59.2	0.1		
	40.8	A	99.7	3.6		
	40.9	A	108.8	34.8		
	41.3	A	8.1	16.3		
	43.2	A	20.7	13.9		
	44.1	A	364	4.1		
	47	A	10.1	1.8		
	50	A	21.7	3.6		
	50.1	A	11	0.6		
	50.1	A	51.4	90.2		
	52.9	A	9.5	10.3		
	53	A	51	37	N	Intra-op GT fx***
	53.7	A	11.2	0.6		
	55.1	A	17.2	6.6		
	55.6	A	24	2		
	56.1	A	7.9	13.1		
	59.6	A	34.6	4.3		
	61.2	A	127.6	3.6		
	62.3	A	28.9	97.3		
	63.6	A	14.8	7.8		
	65.5	A	12.2	0.7		
	66.6	F/A	7.4	4.5		
	74.4	A	6.7	9.8		
	78.3	A	10.1	50.2		
Average	48.5		59.9	22.4

No revisions were performed for the loosening of either the acetabular or femoral component. No patient in the cohort sustained a dislocation, and all implants were stable without evidence of radiographic loosening at the final follow-up. In total, four patients had complications, with three (9.7%) requiring subsequent revision at a mean of 3.6 years after primary post-traumatic THA.

One of these patients sustained an intra-operative greater trochanter fracture. This did not destabilize the femoral implant and was treated conservatively, without additional fixation or modification of standard post-operative protocol following THA.

The second patient underwent metal-on-metal THA 16 years after his index acetabular surgery. He presented four years post-operatively complaining of mild groin pain with elevated blood cobalt and chromium ion levels. He had a normal white blood cell count, erythrocyte sedimentation rate, and c-reactive protein levels, as well as a negative hip aspiration. Intraoperatively, no pseudotumor or metallosis was noted, and minimal abductor heterotopic ossification was debrided. The patient’s symptoms improved after revision to metal-on-polyethylene THA, and he achieved normalization of metal ion levels.

The third patient underwent revision due to persistent pain in the groin region that was attributed to the overhang of the acetabular shell. Inflammatory workup including aspirate was negative, so this was revised, and the patient’s symptoms resolved.

The final patient presented over four years after primary THA with severe groin pain, fevers, and chill and underwent two-stage revision THA. His symptoms resolved following revision THA, without further complication.

## Discussion

Total hip arthroplasty after ORIF is a technically demanding procedure with historically inferior results when compared to primary THA. The aim of this paper has been to determine whether the use of a Hardinge approach during THA in patients who have previously had acetabular ORIF through a posterior, Kocher approach, would reduce rates of dislocation. In this case series of 31 patients, there was a 0% dislocation rate, and 0% incidence of sciatic nerve injury, at a mean follow-up of 22.4 months.

Increased time of the procedure, increased blood loss, and greater overall complication rates are well-established risks of THA after acetabular ORIF when compared to primary THA for nontraumatic arthrosis [3,6,11,12]. However, few studies report on the dislocation rate for THA following acetabular ORIF. Literature reports dislocation rates after THA for post-traumatic arthrosis up to 11%, significantly higher than expected for a primary THA for non-traumatic arthrosis [7]. Unfortunately, these studies are heterogeneous regarding the surgical approach used for THA, and unclear whether the prior acetabular fracture was treated with ORIF (Table 2).

**Table 2:** Approaches and dislocation rates of the studies reviewed *Approaches; AL - Anterolateral, PL - Posterolateral, T-T - Trans-trochanteric, DL - Direct Lateral, THA - Total hip arthroplasty, ORIF - Open reduction internal fixation

Author	Number of THA	Number with prior ORIF	Approach*	Mean follow-up (years)	Dislocation rate	Dislocated THA comments
Khurana et al. [6]	1,199	22 (2%)	-	-	5%	Unknown approach
Morison et al. [7]	74	58 (78)	All Posterior	8	11%	
Bellabarba et al. [11]	30	15 (50%)	3 AL, 3 TT, 9 PL	5.25	0%	
Lizaur-Utrilla et al. [12]	24	9 (38%)	All AL	8.4	4%	Unknown ORIF status
Ranawat et al. [13]	32	24 (75%)	-	4.7	9%	Unknown approach or ORIF status
Stibolt et al. [14]	-	270	-	-	6.20%	Systematic review
Moon et al. [15]	37	37 (100%)	All PL	6.6	8.10%	
Makridis et al. [16]	654	473 (78%)	247 AL, 45 T-T, 236 PL	3.9	4.40%	Systematic review, unknown approach or ORIF status
Weber et al. [17]	66	66 (100%)	19 AL, 36 T-T, 11 PL	9.6	5%	Unknown approach
Huo et al. [18]	21	7 (33%)	All AL	5.42	5%	Unknown ORIF status
Berry et al. [19]	34	15 (44%)	25 AL, 4 TT, 5 PL	10***	3%	Unknown approach or ORIF status
Zhang et al. [20]	55	32 (58%)	2 DL, 28 PL, 2 Combined **	5.33	2%	Unknown approach or ORIF status
Chiu et al. [21]	56	56 (100%)	21 AL	10	-	5% revision rate, unknown dislocation rate
Busch et al. [22]	46	46 (100%)	All DL	4.5	-	1 revision due to recurrent instability

Our literature review of 14 studies that examined THA after acetabular fracture, which included two systematic reviews, had only three that drew direct conclusions regarding dislocation risk with a particular approach after THA for post-traumatic arthrosis after an operatively managed acetabular fracture [6,7,11-22]. Bellabarba et al. reported on 15 of 30 THAs performed after prior acetabular ORIF. Three patients had an anterolateral approach for their THA, three trans-trochanteric, and nine posterolateral, with no overall dislocations [11]. Morison et al. have reported on the largest series to date, with 58 of their 74 THAs undergoing prior acetabular ORIF. These patients all had THAs performed through a posterior approach, with an overall reported dislocation rate of 11% [7]. Finally, Moon et al. reported on 37 THAs done after acetabular ORIF, all via a posterior approach, with a 9% dislocation rate [15]. Given the variation in reporting of post-traumatic THA outcomes, it is difficult to draw conclusions regarding causal or protective factors to dislocation following THA after prior acetabular ORIF. Although Morison et al. included 22% of their cohort without prior ORIF, unlike Moon et al. with 100% prior ORIF, both studies exclusively utilized the posterior approach, reporting 11% and 9% dislocations rates, respectively. Based on these two studies, the posterior approach for post-traumatic THA after operatively managed acetabular fracture is associated with a ~10% dislocation rate.

**Figure 2:**
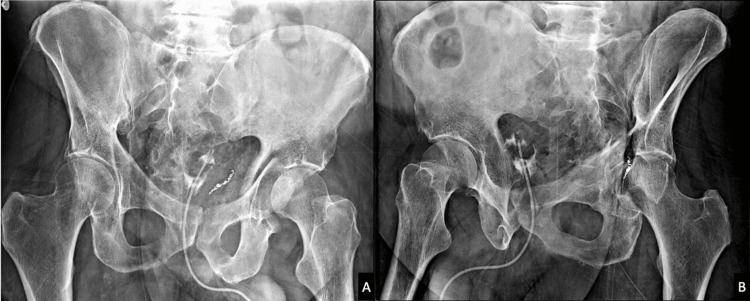
A) Iliac oblique B) obturator oblique radiographs of a 63-year-old male who sustained a column acetabulum fracture after falling from a height

**Figure 3:**
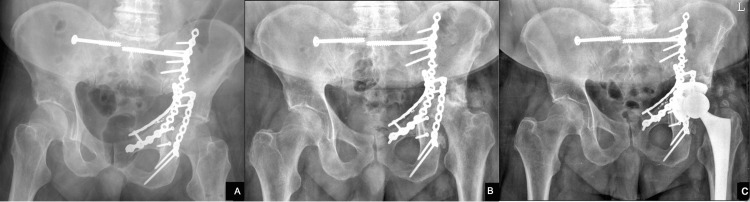
A) Post-op left acetabulum ORIF managed with acute ORIF through Ilioinguinal and Kocher approaches. B) 15 months post-op ORIF, developed advanced post-traumatic arthritis and femoral head avascular necrosis. C) Uncomplicated THA through the Hardinge approach achieved stable painless hip Open reduction internal fixation (ORIF), total hip arthroplasty (THA)

Our case series of 31 patients had all undergone prior a posterior, Kocher-Langenbach approach for acetabular ORIF. The subsequent Hardinge approach was used successfully despite prior soft tissue disruption and existing hardware, without any subsequent episodes of instability (Figures 2A-2B, Figures 3A-3C).

Additionally, given the prior trauma and loss of bone stock that occurs in post-traumatic arthroplasty reconstruction, the ability to obtain good fixation of the acetabular shell has been historically problematic. Cementless fixation of the acetabular shell has a well-documented, high rate of loosening and failure in the literature, up to 32% in the post-traumatic total hip [6,13,18,19]. However, our series did not demonstrate any radiographic acetabular loosening at any follow-up time point. In our experience, as long as both columns are intact without massive bone loss, cementless cup fixation is obtainable.

Our total revision rate of 9.7% is well within the expected revision rate reported in the literature. Despite our success in this case series utilizing the Hardinge approach, it should be taken in the context of its own set of complications, including longer rehabilitation compared to other approaches as the abductors recover. However, studies have shown that all hips tend to arrive at the same functional result when studied at two years and that the post-traumatic hip may even improve further up to four years [3,23].

There are several limitations of this study. First, the short duration of follow-up after THA, just under two years, may underestimate our complication rate. However, the literature suggests that most early dislocations due to component malposition or muscular imbalance declare themselves by two years, with greater than 75% of dislocations occurring within the first year of THA [24-26]. Another limitation is the lack of a standardized functional outcome score such as a Harris hip score. Though all patients were subjectively doing well at their latest post-operative clinic visit, a comparison of pre- and post-operative patient-reported scores could further elucidate the value of performing THA for post-traumatic arthrosis through the second, Hardinge, approach. Further, as the patients selected to receive a Hardinge approach were based on the judgement of a single surgeon, selection bias may be present for cases with more bone stock and less internal hardware present at the time of THA. While this series of patients were able to successfully achieve cementless fixation of the acetabular shell despite the presence of existing hardware, this approach would not be applicable in all existing hardware situations, as the presence of prior hardware can prevent effective fixation of the acetabular shell. As such, it is reasonable to assume that the location of the prior hardware and existing bone stock of the acetabulum are important components of the ability to perform the Hardinge approach. Those acetabular fractures that go on to non-union and lose a significant amount of bone to the point where augments are required are likely to preclude a Hardinge approach as an option. This case series should be taken in the context that the most challenging post-traumatic total hip arthroplasties continue to require a revision posterior approach.

Future studies are needed to further delineate the risks and benefits of the Hardinge approach in this difficult patient population. Prospective studies directly comparing the different approaches for THA, in the setting of prior acetabular ORIF, are needed to make definitive judgements regarding optimal management.

## Conclusions

This series demonstrates a novel paradigm for the management of post-traumatic hip arthrosis following acetabular ORIF via the Kocher-Langenbach approach. Despite prior dissection, all 31 patients in the series received THA via the Hardinge approach, with no subsequent dislocations and no new neurologic injury in our series. The all-cause revision rate of 9.7% is consistent with the prior literature, with no component loosening and only one infection. While the Hardinge approach for post-traumatic total hip arthroplasty cannot be considered a utilitarian approach for post-traumatic reconstruction, it may be an effective way to reduce post-operative instability in this vulnerable patient population.
